# JSA guideline for management of malignant hyperthermia in 2025

**DOI:** 10.1007/s00540-025-03647-y

**Published:** 2026-01-08

**Authors:** Yasuo M. Tsutsumi, Hiroshi Nagasaka, Keiko Mukaida, Yasuko Ichihara, Toshimichi Yasuda, Hirotsugu Miyoshi, The M. H. Management Guideline Working Group of Safety Committee of the Japanese Society of Anesthesiologists

**Affiliations:** 1 Department of Anesthesiology and Critical Care, Hiroshima University, Hiroshima, Japan; 2 Department of Anesthesiology, Saitama Medical University, Saitama, Japan; 3 Department of Anesthesiology, Kikkoman General Hospital, Chiba, Japan; 4 Department of Anesthesiology, Hiroshima Prefectural Rehabilitation Center, Higashihiroshima, Japan

**Keywords:** Malignant hyperthermia, Dantrolene, Volatile anesthetics, Suxamethonium, Genetic testing

## Abstract

Malignant hyperthermia (MH) is a rare, life-threatening inherited disorder triggered by volatile inhalational anesthetics and/or the depolarizing muscle relaxant suxamethonium. In susceptible individuals, calcium release from the sarcoplasmic reticulum in the skeletal muscle becomes abnormally accelerated, leading to a hypermetabolic state. Early signs of MH include unexplained hypercarbia (end-tidal carbon dioxide > 55 mm Hg), tachycardia, and muscle rigidity, particularly in the masseter. Rapid increases in core temperature (> 0.5 °C/15 min, with temperatures often exceeding 40 °C) are typical. With progression, respiratory and metabolic acidosis, arrhythmias, cola-colored urine (myoglobinuria), elevated serum potassium, and tented T-waves may develop, potentially leading to cardiac arrest or multiorgan failure. The Japanese Society of Anesthesiologists’ guidelines for the management of MH in 2025 (Japanese version) emphasize the importance of early recognition and immediate intervention. The essential steps include discontinuing triggering agents, administering intravenous dantrolene (initially 1–2 mg/kg), aggressive cooling of the body, and managing complications, such as hyperkalemia and acidosis. On the basis of international standards, a higher initial dose of dantrolene is recommended. Preoperative assessment of MH risk should include history taking for anesthetic complications, a family history suggestive of MH susceptibility, signs of congenital myopathies, and careful anesthetic planning using non-triggering agents. Genetic testing and muscle biopsy may aid in the diagnosis but are not definitive in all cases. The Japanese translation of these guidelines has been posted on the following website: https://anesth.or.jp/files/pdf/guideline_akuseikounetsu. pdf.

The Japanese Society of Anesthesiologists (JSA) guidelines for the management of malignant hyperthermia (MH) in 2025 were published in Japanese in March 2025. Here, we share an English version of these guidelines to improve medical practice and patient outcomes.

## Objective and target audience

The JSA has issued these guidelines to ensure an understanding of the nature and management of MH. These guidelines describe appropriate anesthesia practices, including testing and treatment, to improve outcomes in patients with MH. This is not intended to outline the medical standards of MH management but to support the care of patients with MH. Therefore, it is recommended that anesthesia care in MH conform to these guidelines and that they be used to promote safe anesthesia care.

## Disclaimer

Many recommendations in this guide are based on expert opinions and case reports in the absence of high-quality evidence because MH is a relatively rare condition. Accordingly, the lack of high-level evidence is a major limitation of this guideline, and its appropriateness and effectiveness need to be scientifically assessed in the future. We aim to assist physicians in decision-making in clinical settings, and the recommendations herein are not intended to be compulsory. The guidelines should be applied with modifications to meet on-site conditions and with limitations according to clinical judgment. In addition, these guidelines do not provide medical care based on their content, nor do they guarantee a positive prognosis.

## Points of revision from the 2016 version

These guidelines were revised with reference to the latest literature and international standards. They recommend an increased initial dantrolene dose based on international literature; moreover, details regarding administration frequency have been included. For brevity, unnecessary sections on symptomatic treatment were removed. Information on genetic testing was included, reflecting a recent increase in its use. Moreover, the references have been updated to the most recent sources. These revisions allow for diagnosis and treatment based on the latest information. These guidelines are expected to serve as a valuable reference for clinical practice.

### Position of the JSA guideline in the context of global MH recommendations

These guidelines are generally aligned with major international recommendations regarding the early recognition of MH, prompt administration of dantrolene, and strict use of non-triggering agents in patients with known or suspected MH. With respect to dantrolene administration, the European Malignant Hyperthermia Group (EMHG) recommends an initial dose of 2–2.5 mg/kg [1–3]. While emphasizing the importance of administering an adequate dose from the outset in Japanese clinical practice, these guidelines present a dosing strategy consistent with the package insert approved in Japan (1 mg/kg).

In the EMHG guidelines, specific requirements for the immediate availability of dantrolene are provided, such as 36 immediately accessible vials and an additional 24 vials obtainable within 1 h [4]. By contrast, this guideline adopts a flexible approach and does not specify a particular stock quantity, allowing each institution to determine its preparedness based on local circumstances.

## Basis of this guideline

Most anesthesiologists rarely encounter MH during routine practice. However, the chances of recovery worsen unless appropriate treatment is administered quickly, because progression occurs extremely rapidly. Therefore, we must gain appropriate knowledge of MH and put it into practice to ensure that intensive care is administered rapidly. Rapid treatment should be started by giving a sufficient dose of dantrolene without waiting for a definitive diagnosis, because early decision-making and treatment are essential. These guidelines present the basis for early therapy and further treatment of hyperthermia with hypermetabolic states and a simple algorithm for management.

### Epidemiology of MH

The incidence of MH is 0.18–3.9 cases per 100,000 general anesthesia procedures [5, 6]. Since the 1960s, more than 600 MH cases have been reported in Japan. Over half of these cases involved individuals aged < 18 years, with a male-to-female ratio of approximately 3:1 [7, 8]. MH is a hereditary muscle disorder, and the frequency of individuals with a latent predisposition (carriers of a causative gene variant) is substantially higher than the incidence of clinical episodes, ranging from approximately 1 in 856 to 1 in 1075 [9, 10]. The mortality rate of MH declined markedly from 70%–80% in the 1960s to 0%–18.2% in 2010. Moreover, among patients treated with dantrolene, the mortality rate is ≤ 10% [8].

### MH pathophysiology

In individuals predisposed to MH, the mechanism of calcium ion (Ca^2^⁺)-induced Ca^2^⁺ release (CICR) from the sarcoplasmic reticulum (SR), which is the Ca^2^⁺ reservoir within skeletal muscle cells, into the cytoplasm is abnormally accelerated.

The initial signs of MH include an unexplained increase in end-tidal carbon dioxide (ETCO_2_ > 55 mmHg), unexplained tachycardia, and muscle rigidity, including masseter muscle rigidity (difficulty in mouth opening). This is caused by abnormally elevated intracellular Ca^2^⁺, which triggers a hypermetabolic state in skeletal muscle cells. Additionally, rapid temperature increases (> 0.5 °C/15 min) may be observed, with core body temperature potentially exceeding 40 °C. Severe respiratory and metabolic acidosis, cardiac arrhythmias, and cola-colored urine (myoglobinuria due to muscle breakdown) can also occur. Serum potassium levels may become elevated and electrocardiographic findings may reveal peaked T-waves, which can progress to cardiac arrest. Moreover, complications such as disseminated intravascular coagulation (DIC) and renal failure may occur. Even if the patient survives an acute episode, long-term sequelae such as muscle dysfunction and impaired consciousness may persist.

**Fig. 1:**
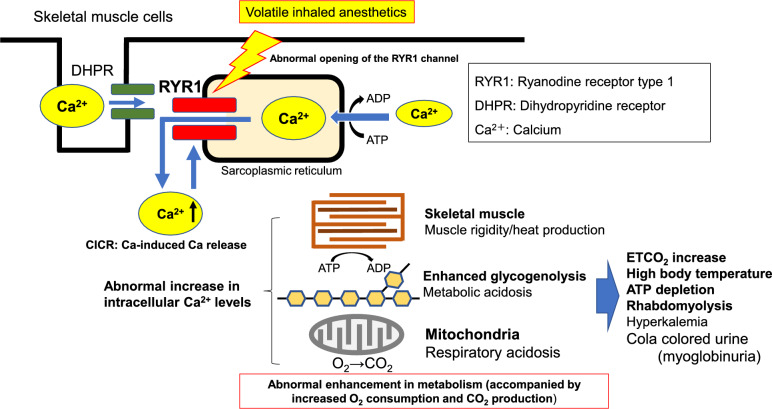
Mechanism of Malignant Hyperthermia (MH): Disruption of the Ca.^2+^ regulation mechanism in skeletal muscle cells. Adapted from JSA Guideline for Management of Malignant Hyperthermia 2025 (Japanese version) (https://anesth.or.jp/files/pdf/guideline_akuseikounetsu.pdf)

All volatile inhaled anesthetics, including sevoflurane and desflurane, as well as depolarizing muscle relaxants such as suxamethonium, are known to trigger MH (Fig. 1). Volatile inhaled anesthetics enhance the rate of Ca^2+^ release from the SR into the cytoplasm. In predisposed individuals, the CICR rate is abnormally accelerated, surpassing the rate of Ca^2^⁺ reuptake into the SR, resulting in an uncontrollable elevation of intracellular Ca^2^⁺ concentration (Fig. 1). This dysregulated Ca^2^⁺ homeostasis is a fundamental pathophysiology of MH. Sedatives and opioids can be safely used in patients predisposed to MH. Remimazolam is reportedly safe in this population [11]. Various environmental factors, such as hot and humid environments, physical exertion, and stress, can enhance the Ca^2+^ release rate from the SR. Therefore, careful monitoring under such conditions is warranted [12, 13].

Abnormally elevated intracellular Ca^2^⁺ in skeletal muscle cells induces sustained pathological muscle contractions, resulting in excessive ATP consumption. Furthermore, the energy-dependent uptake of Ca^2^⁺ into the SR further increases ATP demand, leading to significant heat production. Collectively, these processes contribute to elevated oxygen consumption and excessive CO₂ generation (Fig. 1).

Several proteins are involved in the regulation of Ca^2+^ levels in skeletal muscle cells. Among these, type 1 ryanodine receptor (RYR1) plays a central role in mediating Ca^2^⁺ release from the SR into the cytoplasm and has been strongly implicated in MH pathogenesis (Fig. 1). Additionally, numerous other proteins participate in Ca^2^⁺ homeostasis, and the dihydropyridine receptor has been identified as a potential cause of MH [14].

Sustained elevation of intracellular Ca^2^⁺ levels in skeletal muscle cells leads to a rapid increase in core body temperature. Tissue hypoxia leads to metabolic acidosis, followed by damage to and degradation of muscle cell membranes, resulting in the leakage of intracellular contents into the bloodstream. Consequently, blood levels of potassium and lactic acid, as well as skeletal muscle contents such as myoglobin and creatine kinase (CK), are elevated [15].

Dantrolene acts on the Ca^2+^ release channels (RYR1) in the SR of skeletal muscle cells to suppress Ca^2+^ release [16, 17]. Unlike typical muscle relaxants, dantrolene does not block postsynaptic acetylcholine receptors; instead, it exerts its effects on the skeletal muscle cells. By acting on RYR1 in skeletal muscle cells, it inhibits Ca^2+^ release into the cytoplasm. At therapeutic concentrations (approximately 10 µM), dantrolene has minimal impact on normal skeletal muscle contraction, while effectively suppressing abnormal Ca^2+^ release states such as those seen in MH [18]. This helps limit the excessive energy consumption in skeletal muscle cells under MH conditions. Although the exact mechanism of action remains unclear, direct effects on RYR1 and the indirect effects of other factors may be involved [19]. The onset of action of intravenous dantrolene is approximately 5 min, with a half-life of 5–6 h [20].

### Managing intraoperative and postoperative MH

**Fig. 2:**
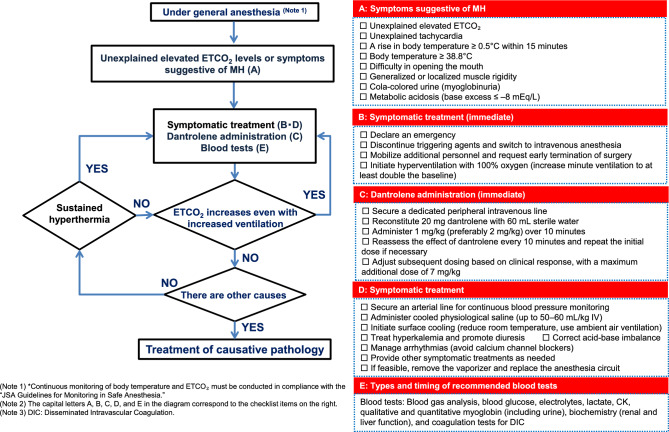
Management algorithm for malignant hyperthermia (MH). Adapted from JSA Guideline for Management of Malignant Hyperthermia 2025 (Japanese version) (https://anesth.or.jp/files/pdf/guideline_akuseikounetsu.pdf)

In patients with symptoms suspected of MH during general anesthesia, implementation of the treatment algorithm outlined in Fig. 2 is recommended. The sequence and frequency of MH symptoms vary by case, making clinical judgment critical in a clinical setting.

Symptoms suggestive of MH include unexplained elevation or high levels of ETCO_2_, unexplained tachycardia, a temperature increase of ≥ 0.5 °C within 15 min, hyperthermia (≥ 38.8 °C), difficulty in opening the mouth, muscle rigidity, cola-colored urine (myoglobinuria), and metabolic acidosis (base excess [BE] ≤ –8.0) [21–23]. Specifically, dantrolene administration is indicated in the presence of early symptoms (increased ETCO_2_ despite increased minute ventilation, unexplained tachycardia, hyperthermia, or muscle rigidity). In such cases, an “Emergency of MH” should be declared, and the measures outlined in Fig. 2 should be promptly implemented.

**Fig. 3:**
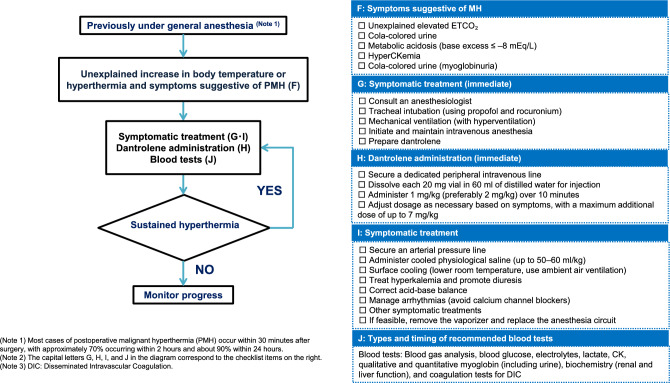
Management Algorithm for postoperative malignant hyperthermia (PMH). Adapted from JSA Guideline for Management of Malignant Hyperthermia 2025 (Japanese version) (https://anesth.or.jp/files/pdf/guideline_akuseikounetsu.pdf)

Notably, MH can also occur after anesthesia [24]. The time to onset of MH symptoms varies widely across cases; however, most cases occur within 40 min of surgery [24]. Therefore, careful monitoring of the postoperative conditions is important (Fig. 3).

## MH crisis treatment procedure (Fig. 2)

1. Discontinue the administration of triggering agents, such as volatile inhaled anesthetics or suxamethonium, and switch to intravenous anesthesia and non-depolarizing muscle relaxants.
2. Declare an emergency, call for additional assistance, and request that the surgeon promptly complete the surgery. All members of the surgical team must recognize that the patient’s condition requires a coordinated team response.
3. Use high-flow pure oxygen (≥ 10 L/min) to perform hyperventilation (set minute ventilation to at least double the normal rate) to reduce the anesthetic concentration in the breathing circuit [25].
4. Prepare dantrolene via dissolution. Because many facilities and operating rooms may not stock large quantities of dantrolene, securing a sufficient supply for a total dose of up to 7.0 mg/kg is necessary.
5. Administer dantrolene via a dedicated large-gauge peripheral intravenous line. Dissolve each 20-mg dantrolene vial in 60 mL of distilled water for injection, shake until clear, and administer at least 1.0 mg/kg (based on actual body weight) over approximately 10 min. Preferably, administer 2.0 mg/kg over approximately 10 min [4]. Repeat administration as needed until there is a decrease in ETCO₂ and core body temperature and improvement in muscle rigidity. The evaluation is performed every 10 min until MH symptoms resolve. The Japanese package insert specifies a maximum of 7.0 mg/kg; however, Western guidelines recommend a dose exceeding 10 mg/kg if dantrolene remains effective [4]. As these guidelines do not set an upper limit for dantrolene dosage, it should be administered in sufficient quantities until clinical symptoms improve.
6. Secure an arterial line for blood gas analysis to determine the degree of metabolic acidosis.
7. Administer chilled normal saline intravenously to reduce the patient’s body temperature (maximum volume: 50–60 mL/kg).
8. Lower the ambient room temperature, and actively cool the patient’s body surface using room-temperature airflow. Discontinue systemic cooling once the patient’s core temperature drops below 38 °C.
9. Treat hyperkalemia as needed. Consider glucose–insulin therapy, calcium gluconate, and sodium bicarbonate administration. Target forced diuresis using furosemide (target urine output: 1.0 mL/kg/h).
10. Treat metabolic acidosis as appropriate and consider the administration of sodium bicarbonate. If the CK levels are elevated or cola-colored urine (myoglobinuria) is present, sodium bicarbonate is administered to promote urinary alkalinization.
11. Manage arrhythmias as clinically indicated. Calcium channel blockers should generally be avoided because their concomitant use with dantrolene may cause cardiac arrest [26]. Alternative agents such as amiodarone or beta-blockers should be considered.
12. Provide other supportive or symptomatic treatments, as clinically indicated.
13. If possible, remove the vaporizer and replace the anesthesia circuit. However, this is not mandatory, because additional personnel and time are required.
14. To assess the patient’s condition, perform the following blood tests: arterial blood gas analysis; blood glucose, electrolytes, lactic acid, CK, qualitative and quantitative myoglobin (including urine) levels; biochemical tests (renal and liver function); and coagulation tests to diagnose DIC. As recurrence may occur after the onset of MH, appropriate diagnostic tests should be conducted as needed, and the patient should be monitored for at least 24 h [27, 28].
15. Clinical improvement in MH can be assessed by observing trends, such as decreasing or normalized ETCO_2_ levels, stabilization of heart rate or fewer arrhythmias, return to normothermia without the need for active cooling, and resolution of muscle rigidity.

### Diagnosing MH

#### Clinical diagnosis

If symptoms and signs of MH are present, a diagnosis should be made clinically and treatment must be initiated immediately. Clinical signs suspicious for MH include unexplained elevations in ETCO_2_, tachycardia of unknown origin, a rapid increase in body temperature (≥ 0.5 °C within 15 min), hyperthermia (≥ 38.8 °C), difficulty in opening the mouth, muscle rigidity, cola-colored urine (myoglobinuria), and metabolic acidosis (BE ≤ –8.0) [21–23]. Persistently elevated ETCO₂ despite increased minute ventilation, an early hallmark of MH, along with unexplained tachycardia, hyperthermia, and generalized muscle rigidity are key indicators of immediate dantrolene administration. In such cases, an emergency due to suspected MH should be declared without delay, and the measures outlined in Fig. 2 must be promptly initiated.

#### Definitive diagnosis of MH

Currently, muscle biopsy and genetic testing are the two main methods used for the definitive diagnosis of MH.

Two well-established methods are available in Europe and the United States for diagnosing MH using muscle bundle specimens obtained via biopsy: the in vitro contracture test (IVCT) and caffeine–halothane contracture test (CHCT). The sensitivity and specificity of IVCT and CHCT are 100% and 94%, respectively, whereas those of CHCT are 97% and 78%, respectively, making it the gold standard for MH diagnosis. However, no Japanese facility has consistently performed these tests [29, 30]. In Japan, the CICR test is performed as a diagnostic muscle biopsy assay for patients suspected of having MH instead of IVCT and/or CHCT. The CICR test is conducted using chemically skinned muscle fibers, and the rate of calcium release from the SR is measured according to Endo’s method [31] at five different calcium concentrations (0, 0.3, 1.0, 3.0, and 10 μM). An accelerated CICR rate is indicative of MH susceptibility. The validity and clinical relevance of the CICR test have been demonstrated in several studies [32, 33]. However, given their invasiveness, there has been a recent decrease in the number of patients who opt for these tests. In addition, the technical complexity of these methods limits the number of facilities that can consistently perform them.

Genetic testing has focused primarily on *RYR1* variants since they were first reported in MH-predisposed individuals [34]. EMHG recognizes dozens of *RYR1* mutations as causative factors of MH [35]. Additionally, variants of *CACNA1S* have been identified as causative factors of MH. Accordingly, if an MH-predisposed individual presents with variants in *RYR1* or *CACNA1S*, they may be identified as MH-susceptible. However, the probability of detecting *RYR1* or *CACNA1S* variants in MH-predisposed individuals is approximately 50–70% [36]. Moreover, some variants are not pathogenic to MH. Therefore, genetic testing has limited diagnostic accuracy. Consequently, efforts are being made to identify and characterize additional gene variants that can be definitively recognized as pathogenic for MH, to improve the diagnostic reliability of genetic testing.

## Perioperative risk assessment and management

### Preoperative assessment and management for patients suspected of MH

#### 1. Surgical history

Obtaining the history of intraoperative and postoperative episodes of hyperthermia, muscle rigidity, cola-colored urine (myoglobinuria), and muscle pain is important. If a history of such episodes is apparent, the patient should be treated as potentially susceptible to MH. MH is a genetic disease with a penetration rate of 40.6% [37]. Even if there is no apparent history of previous anesthesia experience, ruling out MH is inappropriate because it is not necessarily provoked, even if triggering agents are used [37, 38].

#### 2. Past history of heat stroke, exercise-induced rhabdomyolysis, and familial history

Patients with MH reportedly have a family history of heat stroke and/or MH or *RYR1* variants [38, 39]. Similarly, *RYR1* variants have been implicated in exercise-induced rhabdomyolysis, suggesting that their presence indicates MH risk [1, 40].

#### 3. CK level

There is no apparent correlation between serum CK levels and MH susceptibility. However, idiopathic hyperCKemia may indicate susceptibility to MH [1].

#### 4. Congenital myopathies related to MH [41]

Findings suggestive of congenital myopathy, such as delayed motor development (e.g., late-to-start walking), proximal muscle weakness, high palate, scoliosis, ptosis, and joint contractures, may be related to MH and should be considered cautiously. Congenital myopathies associated with *RYR1* variants and susceptibility to MH include central core disease, multi-minicore disease, and King–Denborough syndrome.

### Caution in planning safe anesthesia

1. Selection of safe anesthetics and anesthesia protocol, and prophylactic administration of dantrolene.

2. Ca^2+^ channel blockers.

Antagonists of L-type Ca channels reportedly increase intracellular Ca^2+^ levels in skeletal muscle cells [26]. Ca^2+^ blockers such as verapamil should not be used along with dantrolene because hyperkalemia and profound hypotension may occur with this combination [42].

3. Preparation of intravenous dantrolene and distilled water for dissolution

The initial dose of dantrolene at 1.0 mg/kg (based on the patient’s actual body weight) should be prepared along with the necessary amount of distilled water (60 mL per 20-mg vial) and kept readily available.

4. Preparation of the anesthesia machine [3]

The vaporizer and should be removed and the breathing circuit, bag, and carbon dioxide absorbent should be replaced with new ones. The anesthesia machine should be flushed with high-flow gas (≥ 10 L/min of 100% oxygen, air, or a mixture) to wash out residual volatile inhalational anesthetics. The time required for flushing varies by machine; however, it tends to be longer in newer models, which typically require 60–100 min with 10 L/min pure oxygen [3]. High-flow gas should be continued until the machine is used and avoid placing it in standby mode. Reducing the flow rate can cause a rebound effect, which increases anesthetic concentration. A ventilator without a volatile anesthetic vaporizer can be used as an alternative to anesthesia machines.

5. Maintaining anesthesia and postoperative care

Adequate monitoring and appropriate postoperative care are essential. Monitor ETCO_2_ and core temperature continuously. Caution is necessary because no causative anesthetic drugs were reportedly used in 7 of the 477 cases of MH in a previous study [21]. If suspicious findings are observed in MH-susceptible patients during anesthesia, dantrolene should be administered intravenously. In the absence of suspicious MH findings, standard postoperative management is considered safe and appropriate [3].

### Counseling patients and their family members after MH diagnosis

#### Important explanatory points

1. MH is a pharmacogenetic disorder of the skeletal muscles that presents as a hypermetabolic response to inhalational anesthetics and depolarizing muscle relaxants, potentially leading to death [5].
2. Patients are free from any signs of MH in their everyday activities, with the exception of hard physical labor.
3. Biochemical, hematological, and physical tests lack significant sensitivity and specificity for diagnostic use.
4. If MH or MH susceptibility has been determined in blood relatives, treat the patient as MH susceptible and notify relatives of the possibility of MH.
5. Exercise-related stress and heat stroke are potential triggers for MH.

#### Include the following items concerning future anesthesia

1. Prescribing dantrolene before anesthesia is not recommended because it causes muscle weakness [3].
2. Local anesthetics and regional anesthesia including subarachnoidal and epidural anesthesia are safe. Intravenous administration of sedatives is preferable to reduce anxiety and stress [1, 3, 5, 43].
3. Inhalational anesthetics and depolarizing muscle relaxants should be avoided when receiving medical treatment that requires general anesthesia. Intravenous anesthetics are considered safe.
4. These patients are recommended to receive medical treatment at facilities where dantrolene is available.
5. Genetic testing and muscle biopsy can be used to determine susceptibility to MH, and a definitive diagnosis is recommended. The probability of confirming MH susceptibility through testing is approximately 50%–70%; even when the results of muscle biopsy and genetic testing are negative, this is not sufficient to rule out MH.
